# Supplementary material for: An Evaluation of Phylogenetic Workflows in Viral Molecular Epidemiology
Source: Viruses. 2022 Apr 8;14(4):774. doi: 10.3390/v14040774 (PMC9032411; doi:10.3390/v14040774)
Supplement: Supplementary file 1 [file viruses-14-00774-s001.zip › viruses-1643227-supplementary.pdf]

Supplementary Materials

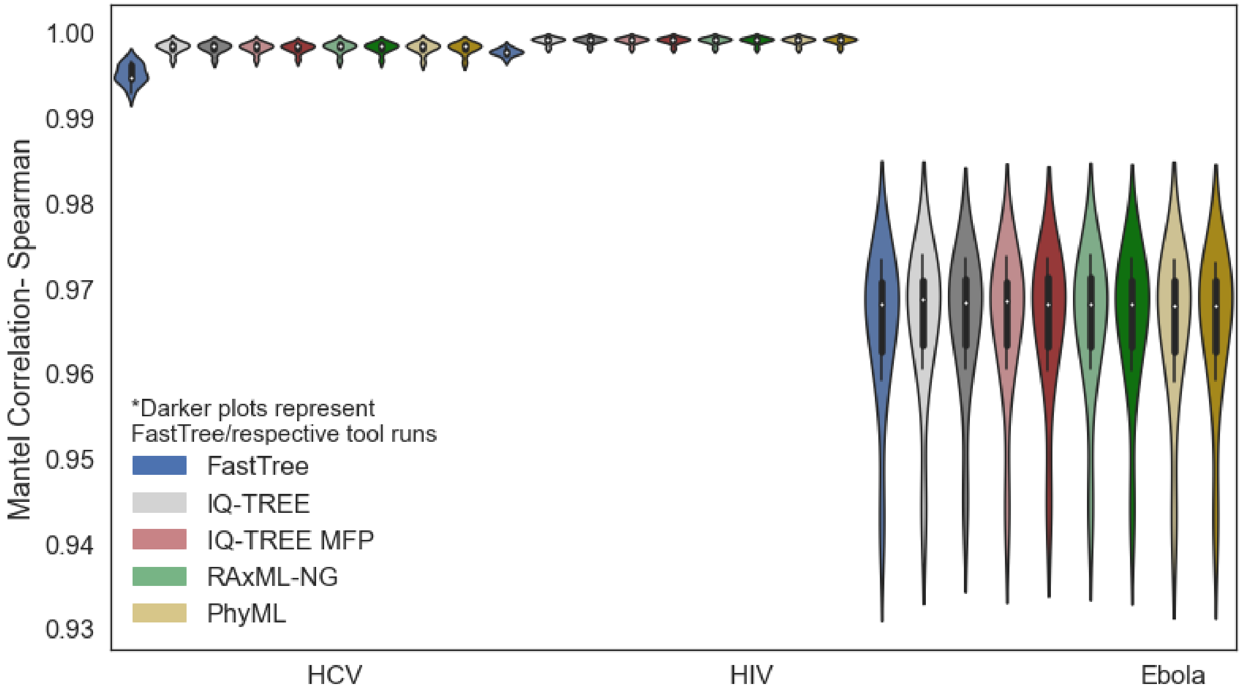

Supplementary Figure S1. Violin plots of the Spearman Mantel Correlations for the patristic distance matrices of phylogenies inferred by FastTree, IQ-TREE, IQ-TREE (MFP), RAxML-NG, and PhyML from 10 simulated replicate datasets of HIV, HCV, and Ebola. Phylogenies which result from optimizing branch lengths along FastTree topology are also included.

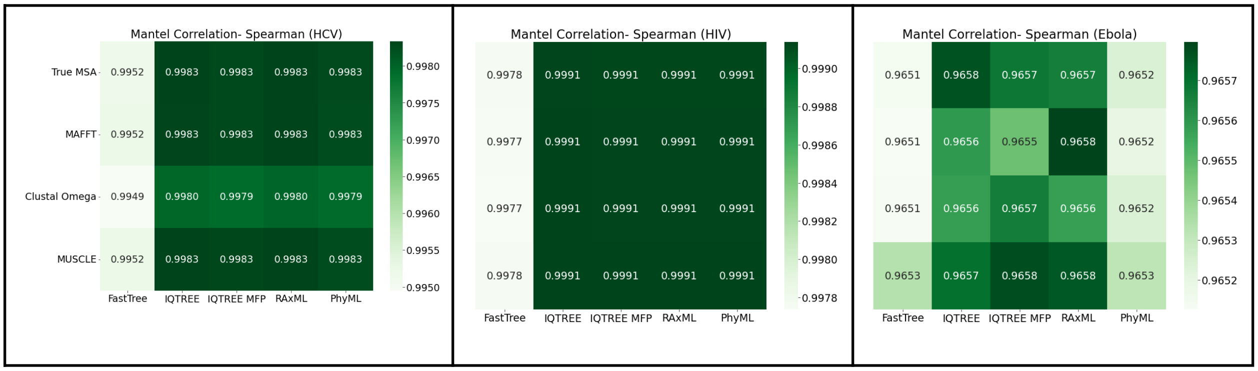

Supplementary Figure S2. Heat maps comparing the average Spearman Mantel Correlations for patristic distance matrices of phylogenies inferred with FastTree, IQ-TREE, IQ-TREE (MFP), RAxML-NG, and PhyML from the MAFFT, Clustal Omega, MUSCLE, and true multiple sequence alignments. Values shown are the average of 10 replicate datasets for each virus.

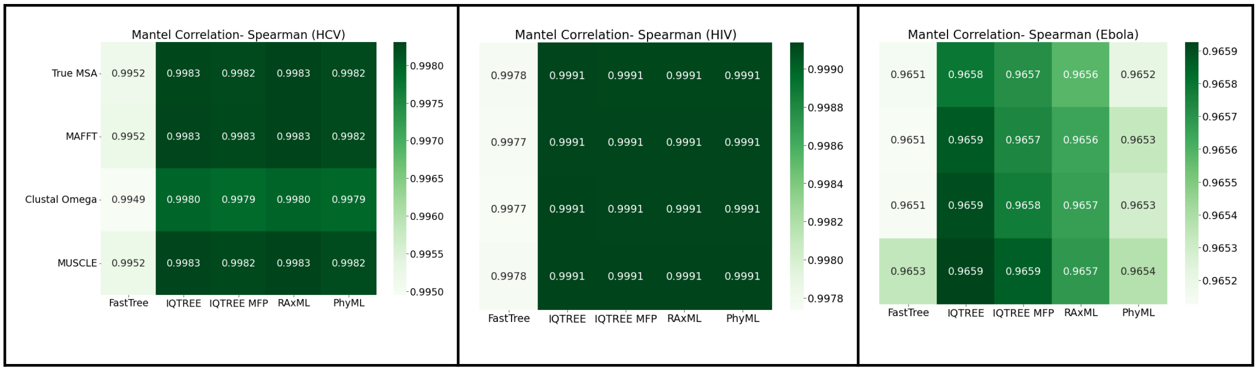

Supplementary Figure S3. Heat maps comparing the Spearman Mantel Correlations for patristic distance matrices of FastTree topologies inferred from the MAFFT, Clustal Omega, MUSCLE, and true multiple sequence alignments with branch lengths optimized by IQ-TREE, IQ-TREE (MFP), RAxML-NG, and PhyML. Values shown are the average of 10 replicate datasets for each virus.
